# Editorial: Impact of vestibular dysfunction studies on space flight health challenges

**DOI:** 10.3389/fneur.2025.1758473

**Published:** 2025-12-16

**Authors:** James R. Lackner, Pierre Denise, Scott J. Wood

**Affiliations:** 1 Ashton Graybiel Spatial Orientation Laboratory, Brandeis University, Waltham, MA, United States; 2 INSERM, COMETE U1075, CYCERON, CHU de Caen, University of Caen Normandy, Caen, France; 3 Neuroscience Laboratory, NASA Johnson Space Center, Houston, TX, United States

**Keywords:** adaptation, performance, prediction, spaceflight, vestibulopathy

## Introduction

The use of patients and terrestrial analogs to examine how vestibular function might influence spatial perception and motor output during spaceflight spans the history of space health research (1, 2). One goal of this topic was to translate insights drawn from studies of individuals without bilateral vestibular function to how they would perform in spaceflight, where otolith signals no longer provide a sense of spatial orientation. Five papers relate to the topic, two of which provide direct support for including parastronauts without vestibular function in future missions. Their immunity to motion sickness and resistance to illusions typically evoked in dynamic and weightless aerospace environments would be highly advantageous in operational conditions. Additional papers address other spaceflight analogs, such as head down bedrest (HDBR) and sustained Gx centrifugation, as well as a review of velocity storage and motion sickness susceptibility, that together highlight critical vestibular challenges in space.

## Case for bilateral vestibulopathy parastronauts

Ramsburg et al. reviewed how governmental and commercial space agencies are evaluating the feasibility of having parastronauts participating in future missions (3). The present paper summarizes studies that Graybiel (1) conducted early in the American space program with students from Gallaudet University with bilateral vestibulopathy (BVP). These studies demonstrated the advantages that BVP individuals would have in aerospace conditions and artificial gravity environments over astronauts with full vestibular function.

BVP individuals have been proven to be immune to motion sickness in every motion context yet tested including parabolic flight, ships in storms, exposure to Coriolis forces, drop towers and many other provocative contexts. They do not perceive the oculogravic and oculogyral illusions associated with exposure to linear acceleration and angular acceleration, respectively. In the weightless phases of parabolic flight, they do not experience inversion illusions unlike subjects with full vestibular function. In rotating artificial gravity environments, the BVP individuals adapt readily and without experiencing motion sickness to the Coriolis forces that perturb head and body movements, unlike control subjects who adapt much more slowly and to lesser force levels. These characteristics of BVP individuals would give them an advantage in adapting to spaceflight in general and to reduced gravity conditions on the Moon (0.16 G) and Mars (0.38 G) and to future rotating artificial gravity space vehicles.

Clement et al. is of exceptional interest because it is the first to contrast the performance of astronauts on return from prolonged spaceflight with BVP individuals on tasks mapping different aspects of postural control and attention (sit-to-stand, walk-and-turn, tandem walking, reaction time, and duration estimation). The BVP groups' performance on the postural tasks was stunningly like that of returning astronauts on Day 1, their performance on the cognitive tasks was comparable to that of the astronauts' inflight results. Given the large sample (28 astronauts, 30 BVP subjects), the results are compelling. They also raise the possibility that additional tests of BVP subjects on other challenging tasks could provide predictions of other situations in which astronauts could be vulnerable inflight and postflight.

## Additional spaceflight analogs

Tays et al. utilized HDBR as an analog to mimic the headward shifts of blood that occur in weightlessness and other deconditioning effects. In this study, a broad range of tasks were degraded both in space flight and in prolonged HDBR. The goal was to determine over 60 days of HDBR whether passive centrifugation with two Gs at the feet, 1G at the center of mass and 0.3G at the head for brief periods, 30 min, could prevent the deterioration of cognitive performance elicited by HDBR. Two experimental groups one with 30 min per day exposure and one with six 5-min exposures per day, were tested along with control subjects without centrifugation. There were no significant performance differences among the groups. The discussion and conclusion sections are valuable in highlighting the limitations imposed by the brief periods of centrifugation, and the value of artificial gravity in a variety of other performance tasks.

Lonner et al. describe a ground-based procedure (SIC, sickness induced by centrifugation) to study aspects of the vestibular related disruptions astronauts experience on return from prolonged spaceflight. Twenty subjects were exposed on 1 day to two Gx centrifugation for 1 h, and on another day to 1 h of supine rest, in counterbalanced order. Afterwards, they underwent various dynamic and static tilt profiles for pitch and for roll, estimating their amplitudes. The results showed significant underestimations for pitch (33%), but not for roll, after two Gx exposure compared with their supine control condition. The contribution of the saccules and utricles in giving rise to these effects is delineated and justified. A valuable feature of the paper is the discussion of the difficulties faced in trying to develop valid ground-based analogs of post-spaceflight disturbances.

## Velocity storage and motion sickness

Maruta provides a comprehensive overview of the phenomenon of velocity storage of nystagmus, wherein eye movements last considerably longer than the sensory signals that elicit them. Importantly, he points out that nystagmus is not only elicited by visual and by vestibular semicircular canal activation but also by proprioceptive stimulation, off vertical body rotation, and sinusoidal motion of the whole body. A key feature of the paper is a chart showing how exposure to weightlessness, and specific vestibular and cerebellar lesions, affect the properties of velocity storage. Velocity storage magnitude is causally related to susceptibility to motion sickness and a wide-ranging review of the circuitry involved, including the nodulus and uvula of the cerebellum, is provided. The review emphasizes that we still lack a comprehensive understanding of how the multiple contributions to velocity storage and to motion sickness susceptibility are interrelated. Individuals without vestibular function are immune to motion sickness and thus do not have velocity storage associated with angular acceleration of the head. An implication of the paper is that much might be learned from exploring whether proprioceptive and other non-vestibular signals can evoke velocity storage in such individuals.

## Conclusions

The immunity of BVP individuals to motion sickness would be to their advantage if they were in spaceflight. Many of the adaptations they make to living without vestibular function on Earth would potentially be advantageous in spaceflight. What types of performance decrements they might show postflight compared to astronauts with full vestibular function need to be considered as part of an ongoing risk assessment given increasing opportunities provided by commercial spaceflight (4). In spite of these challenges, we would advocate for the inclusion of BVP parastronauts based on the valuable insight and contributions they can provide for both spaceflight and terrestrial healthcare (5).
